# Polymorphic Variation in *TIRAP* Is Not Associated with Susceptibility to Childhood TB but May Determine Susceptibility to TBM in Some Ethnic Groups

**DOI:** 10.1371/journal.pone.0006698

**Published:** 2009-08-20

**Authors:** Shobana Rebecca Dissanayeke, Samuel Levin, Sandra Pienaar, Kathryn Wood, Brian Eley, David Beatty, Howard Henderson, Suzanne Anderson, Michael Levin

**Affiliations:** 1 Department of Paediatric Infectious Diseases, Imperial College London, London, United Kingdom; 2 Paediatric Infectious Diseases Unit, Red Cross Children's Hospital, School of Child and Adolescent Health, University of Cape Town, Cape Town, South Africa; 3 Institute of Infectious Diseases and Molecular Medicine, Faculty of Health Sciences, University of Cape Town, Cape Town, South Africa; University of Stellenbosch, South Africa

## Abstract

Host recognition of mycobacterial surface molecules occurs through toll like receptors (TLR) 2 and 6. The adaptor protein TIRAP mediates down stream signalling of TLR2 and 4, and polymorphisms in the TIRAP gene (*TIRAP*) have been associated with susceptibility and resistance to tuberculosis (TB) in adults. In order to investigate the role of polymorphic variation in *TIRAP* in childhood TB in South Africa, which has one of the highest TB incidence rates in the world, we screened the entire open reading frame of *TIRAP* for sequence variation in two cohorts of childhood TB from different ethnic groups (Xhosa and mixed ancestry). We identified 13 SNPs, including seven previously unreported, in the two cohorts, and found significant differences in frequency of the variants between the two ethnic groups. No differences in frequency between individual SNPs or combinations were found between TB cases and controls in either cohort. However the 558C→T SNP previously associated with TB meningitis (TBM) in a Vietnamese population was found to be associated with TBM in the mixed ancestry group. Polymorphisms in *TIRAP* do not appear to be involved in childhood TB susceptibility in South Africa, but may play a role in determining occurrence of TBM.

## Introduction

Activation of host inflammatory responses are fundamental to containment of *Mycobacterium tuberculosis* (MTB) following initial infection [1]. The majority of individuals who are exposed to MTB successfully contain the infection and remain asymptomatic but latently infected throughout life. TB disease develops in a minority of those infected, either following the primary infection or due to reactivation of latent disease many years later [2]. Differences in immune response genes are believed to be important in determining whether an individual successfully contains the infection or develops disease [3], [4], [5], but the genes responsible remain largely unknown.

Children not only have an increased risk of developing progressive disease following exposure [6], but have a much greater risk of developing disseminated forms of the disease including TBM [7], [8]. The ability to rapidly activate innate immune responses is likely to be critical in the containment of mycobacteria during primary infection in childhood, as evidenced by the unique susceptibility to mycobacteria in patients with Mendelian defects in interferon gamma (IFNγ) and interleukin 12 (IL-12) pathways [9], [10], [11], [12].

Innate immune recognition of mycobacterial surface molecules including lipoarabinomannan, are believed to occur predominantly through toll-like receptor (TLR) 2 [13] with TLR6 as its co receptor [14], and possibly TLR4 [15]. Binding of the ligand initiates a signalling cascade leading to activation of proinflammatory responses [16]. The TIR-domain-containing adapter like protein (TIRAP) is a cytoplasmic protein which is 221 amino acids in length and is important in both the TLR2 and TLR4 mediated signalling pathways [17], [18]. Binding of the ligand to either receptor leads to the recruitment of several molecules to the receptor including the TIR-domain-containing adaptor molecules such as the myeloid differentiation primary response gene 88 (MyD88) and TIRAP [18]. This complex recruits other molecules including IL-1R kinases 1, 2, 3 and 4 (IRAK1, 2, 3 and 4) and TNF receptor associated factor 6 (TRAF6) [19] which dissociate and bind a further complex that consists of transforming growth factor-β-activated kinase 1 (TAK1) and TAK-1-binding proteins 1, 2 and 3 (TAB 1, 2, 3). TAK1 activates the inhibitor of NFκB kinase (IKK) via IKKγ (also called NEMO). IKK causes the phosphorylation of the inhibitor of NFκB (IκB), which leads to its degradation and thereby allows NFκB to translocate to the nucleus and promote the transcription of proinflammatory genes.

**Table 1:** Sequence variants in *TIRAP* exons and 3′ untranslated region in Xhosa and mixed ancestry populations.

SNP	MAF Xhosa	MAF Coloured	p value
p25 G/C^a^	0.04	0.02	NS
p164 G/A^a^	0.05	0.02	NS
p298 G/A^ab^	0.004	0.007	NS
p303 G/A	0.002	0	NA
p393 C/T^b^	0.03	0.03	NS
p427 C/T^ab^	0.004	0	NA
P539 C/T^a^	0.002	0.04	**<0.001**
P548 G/C^ab^	0.009	0.01	NS
p558 C/T	0.03	0.13	**<0.001**
p589 G/A^a^	0.02	0.03	NS
P655 C/T^b^	0.02	0.01	NS
p760 A/G^b^	0.09	0.09	NS
p820 C/T	0.47	0.2	**<0.001**

Minor allele frequency (MAF) of each SNP is shown in the Xhosa and Mixed ancestry populations (Cases and controls combined). P values were calculated by the Fishers exact test. Significant differences in MAF between the two populations are seen at positions 539, 558 and 820.

a These polymorphisms caused an amino acid change.

b These polymorphisms are novel.

**Table 2:** Frequency of *TIRAP* variants in cases and controls for Xhosa and Mixed ancestry groups.

SNP position		Xhosa			Mixed ancestry		
(Amino acid change)	Genotype	Cases (%)	Controls (%)	p-value	Cases (%)	Controls (%)	p-value
**p25**	**CC**	125 (93)	106 (89)		51 (94)	15 (100)	
**(A9P)**	**CT**	8(6)	12 (10)	0.3	3 (6)	0	0.6
	**TT**	0	0		0	0	
**p164**	**GG**	122 (0.91)	101 (89)		45 (89)	13 (100)	
**(S55N)**	**GA**	12 (9)	13 (11)	0.6	7 (11)	0	0.5
	**AA**	0	1 (0.01)		1	0	
**p298**	**GG**	142 (99)	121 (99)		53 (0.98)	16 (100)	
**(A100T)**	**GA**	1 (1)	1 (1)	1	1 (2)	0	1
	**AA**	0	0		0	0	
**p303**	**GG**	142 (99)	122 (100)		54 (100)	16 (100)	
	**GA**	1 (1)	0	1	0	0	NA
	**AA**	0	0		0	0	
**p393**	**CC**	135 (94)	112 (92)		50 (93)	16 (100)	
	**CT**	8 (6)	10 (8)	0.5	4 (7)	0	0.6
	**TT**	0	0		0	0	
**p427**	**CC**	143 (100)	120 (98)		50 (93)	16 (100)	
**(R143W)**	**CT**	0	2 (2)	0.2	0	0	NA
	**TT**	0	0		0	0	
**p539**	**CC**	142 (99)	122 (100)		51 (94)	14 (88)	
**(S180L)**	**CT**	1 (1)	0	1	3 (6)	1 (6)	0.3
	**TT**	0	0		0	1 (6)	
**p548**	**GG**	141 (99)	119 (98)		52 (96)	16 (100)	
**(R184T)**	**GC**	2 (1)	3 (2)	0.7	2 (4)	0	1
	**CC**	0	0		0	0	
**p558**	**CC**	135 (94)	114 (93)		41 (76)	13 (81)	
	**CT**	8 (6)	8 (7)	0.8	12 (22)	2 (13)	0.5
	**TT**	0	0		1 (2)	1 (6)	
**p589**	**GG**	136 (95)	117 (96)		51 (94)	15 (0.94)	
**(V197I)**	**GA**	7 (5)	5 (4)	0.8	3 (6)	1 (0.06)	1
	**AA**	0	0		0	0	
**p655**	**CC**	138 (97)	118 (97)		52 (96)	16 (100)	
	**CT**	5 (3)	4 (3)	1	2 (4)	0	1
	**TT**	0	0		0	0	
**p760**	**AA**	116 (81)	102 (84)		44 (81)	14 (88)	
	**AG**	26 (18)	19 (16)	0.8	9 (17)	2 (13)	1
	**GG**	1 (1)	1 (1)		1 (2)	0	
**p820**	**CC**	29 (20)	40 (33)		0	0	
	**CT**	63 (44)	48 (39)	0.7	20 (37)	8 (50)	0.4
	**TT**	50 (35)	34 (28)		34 (63)	8 (50)	

The p-values for differences between cases and controls in each ethnic group were calculated by Fishers exact test or the Freeman Halton extension of the Fishers exact test when all three genotypes were present. No significant differences in the frequency of any SNP or genotype was observed between cases and controls. Differences in numbers of samples are due to sequence failures.

**Table 3:** Frequency of SNP combinations in Xhosa and mixed ancestry groups.

Combination number	Genotype	Xhosa		Mixed ancestry	
		Cases	Controls	Cases	Controls
1	No SNP	0.17	0.26	0.13	0.33
2	25GC	0.02	0.06	*	*
3	164GA	0.02	0.02	*	*
4	820CT	0.27	0.17	0.19	0.25
5	820TT	0.11	0.06	*	*
6	25GC+820CT	0.02	0.02	0.02	0.00
7	25GC+820TT	0.01	0.00	*	*
8	164GA+820CT	0.03	0.08	*	*
9	164GA+820TT	0.01	0.01	0.02	0.00
10	164AA+820TT	0.00	0.01	0.02	0.00
11	298GA+820CT	0.01	0.00	*	*
12	298GA+820TT	*	*	0.02	0.00
13	393CT+760AG	0.00	0.01	*	*
14	393CT+820CT	0.02	0.02	*	*
15	393CT+820TT	0.01	0.05	0.04	0.00
16	539CT+820CT	*	*	0.02	0.00
17	539CT+820TT	*	*	0	0.08
18	548GC+820TT	*	*	0.02	0.00
19	551GC+820TT	0.01	0.00	*	*
20	558CT+820CT	0.01	0.03	0.04	0.08
21	558CT+820TT	0.02	0.01	0.08	0.00
22	558TT+820TT	*	*	0.02	0.08
23	589GA+820CT	0.01	0.01	0	0.08
24	589GA+820TT	0.02	0.00	0.02	0.00
25	655CT+820CT	0.00	0.01	*	*
26	655CT+820TT	0.02	0.00	*	*
27	760AG+820CT	0.04	0.04	0.06	0.08
28	760AG+820TT	0.07	0.06	0.08	0.00
29	760GG+820TT	0.01	0.01	0.02	0.00
30	25GC+164GA+820TT	0.01	0.00	*	*
31	25GC+539CT+820CT	*	*	0.02	0.00
32	25GC+558CT+820CT	0.00	0.01	*	*
33	25GC+760AG+820CT	0.01	0.01	*	*
34	164GA+298GA+820TT	0.00	0.01	*	*
35	164GA+558CT+820TT	0.01	0.00	0.04	0.00
36	164GA+589GA+820TT	*	*	0.02	0.00
37	164GA+655CT+820TT	*	*	0.02	0.00
38	164GA+760AG+820CT	0.01	0.00	*	*
39	164GA+760AG+820TT	0.02	0.00	0.02	0.00
40	303GA+539CT+820CT	0.01	0.00	*	*
41	393CT+558CT+820TT	0.00	0.01	0.04	0.00
42	393CT+589GA+820TT	0.01	0.00	*	*
43	393CT+655CT+820TT	0.01	0.00	*	*
44	551GC+589GA+820TT	0.01	0.00	*	*
45	548GC+589GA+820TT	*	*	0.02	0.00
46	558CT+589GA+820TT	*	*	0.02	0.00
47	558CT+760AG+820CT	0.00	0.01	*	*
48	558CT+760AG+820TT	0.02	0.00	*	*
49	589GA+760AG+820TT	0.01	0.01	*	*
50	655CT+760AG+820CT	0.01	0.01	*	*
51	25GC+551GC+589GA+820CT	0.00	0.01	*	*
52	164GA+655CT+760AG+820TT	*	*	0.02	0.00
53	551GC+589GA+655CT+820TT	0.00	0.01	*	*

* Combination not present.

Recent studies on the TIRAP gene have indicated that it may play an important role in susceptibility to infectious diseases. The single nucleotide polymorphism (SNP) 539C→T (S180L) has been associated with protection against several infectious diseases including pneumococcal disease, malaria and TB in Caucasian, African and South American cohorts [20], [21]. This result was not replicated in a subsequent study on different European and African groups [22]. In a Vietnamese cohort a synonymous SNP 558C→T (A186A) was associated with TB meningitis in adults and also with functional impairment of IL-6 production *in vitro* [23].

While these studies suggest that variation in *TIRAP* is potentially important in determining susceptibility to TB and other infections, the inconsistent findings between published data and the different SNPs implicated in each, results in continued uncertainty as to its role in influencing disease occurrence and outcome. One of the reasons for this might be due to the ethnic differences in the groups studied. The majority of studies have focused on the S180L polymorphism that was first implicated in a Caucasian cohort with invasive pneumococcal disease. This polymorphism has a much lower frequency in African populations. Similarly, the A186A SNP, associated with TB meningitis in the Vietnamese population, was not studied in relation to clinical phenotype in the African population.

We set out to investigate the role of sequence variation in *TIRAP* in children from two ethnic groups from South Africa, an area with one of the highest TB incidence rates in the world [6], [24]. We reasoned that genetic differences in the innate immune response would be more important in childhood TB where the inability to contain the organism following initial infection is the usual mechanism of disease. In order to detect not only previously known variants, but also any novel polymorphisms or mutations, we screened the entire open reading frame of the *TIRAP* for sequence variation in cases with childhood TB and healthy controls. In order to understand ethnic differences we studied individuals from both the Xhosa populations and those with mixed ancestry.

## Results

### Polymorphic variation in Xhosa and mixed ancestry groups

We identified thirteen SNPs across the TIRAP gene of which only seven have previously been identified. Two SNPs, the 303C→T and 427C→T were only seen in the Xhosa population and were not found in the smaller mixed ancestry group (Table 1).

The minor allele frequency (MAF) of the majority of SNPs was low (<0.05) with only 760A→G and 820C→T having a higher frequency in both groups and the 558C→T in the mixed ancestry group only (table 1). Three SNPs were significantly different between the two groups. Two of these, the 539C→T (S180L) and 558C→T have been associated with TB disease in previous studies in different ethnic groups [20], [23]. The 820T→C SNP has almost equal allele frequencies in the Xhosa group but the T allele was present in 80% of the mixed ancestry group. The frequency in the Xhosa is similar to frequencies of this SNP found in the PGA African panel of African Americans, whereas that in the mixed ancestry group was similar to the frequency in the PGA European panel as published in the NCBI database (ss8820536) [25]. The CC genotype was not seen in any of the mixed ancestry subjects although it was prevalent in 20% and 30% of the Xhosa cases and controls respectively.

Only five of the 11 SNPs identified in the mixed ancestry TB cases were also found in the controls. However as the number of controls was small and as these alleles were at low frequency this result may be due to sample size differences alone.

### Case Control study

**Figure 1:**
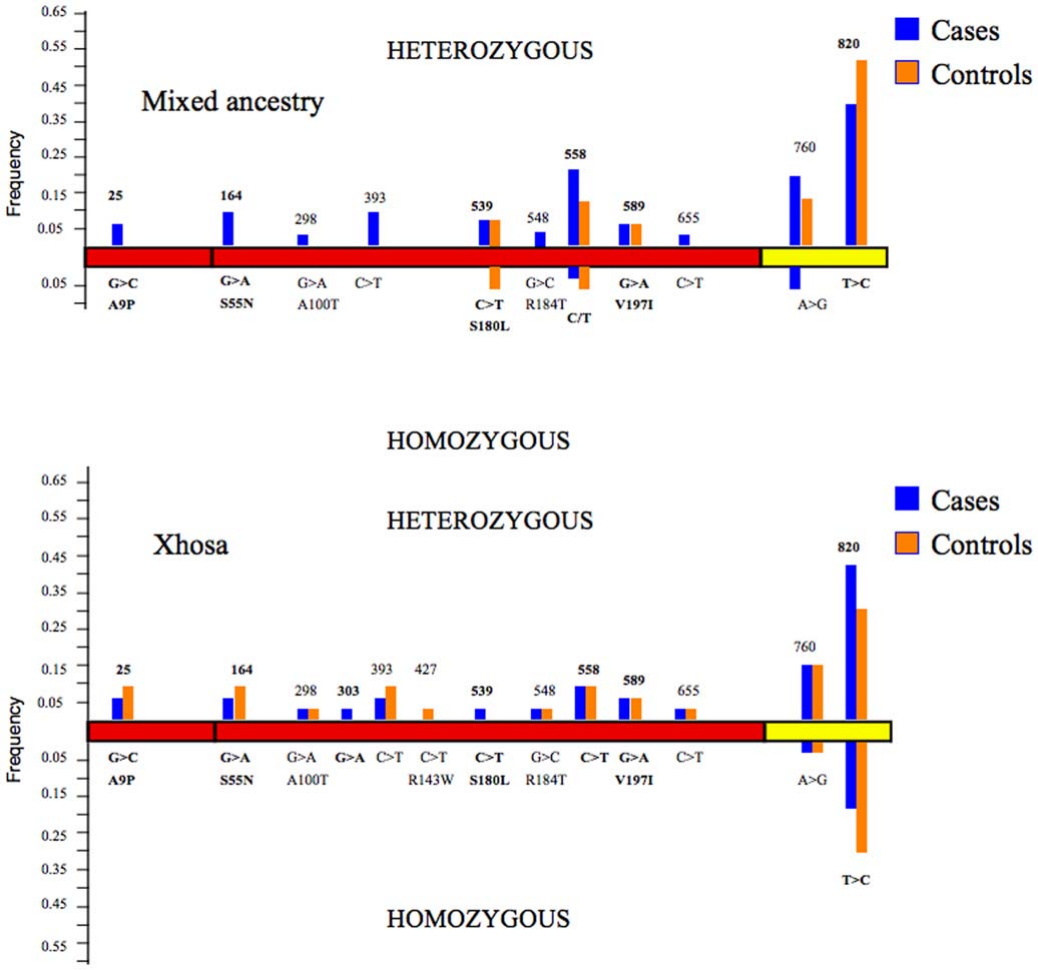
Sequence variation in *TIRAP* in the Mixed ancestry and Xhosa cases and controls. Representation of the opening reading frame of the TIRAP gene with the frequency of SNPs in the A) Mixed ancestry group and in the B) Xhosa group at various positions in the gene. Red blocks represent the coding region and the yellow blocks the 3′ UTR. Bold numbers and letters show published SNPs. Heterozygous SNPs with position in the gene are shown above the gene diagram; homozygous SNPs with base change and amino acid change (if nonsynonymous) shown below the gene diagram.

Having identified SNPs in the coding region in each ethnic group, we next sought to identify any association between individual *TIRAP* variants, or combinations of variants, with disease. Table 2 shows the frequency in cases and controls of each of the identified SNPS, and figure 1 displays the pattern of variation across the gene for both heterozygous and homozygous individuals. There were no statistically significant differences between the frequency of any individual SNP between cases and controls in either cohort.

### Haplotype analysis

**Figure 2:**
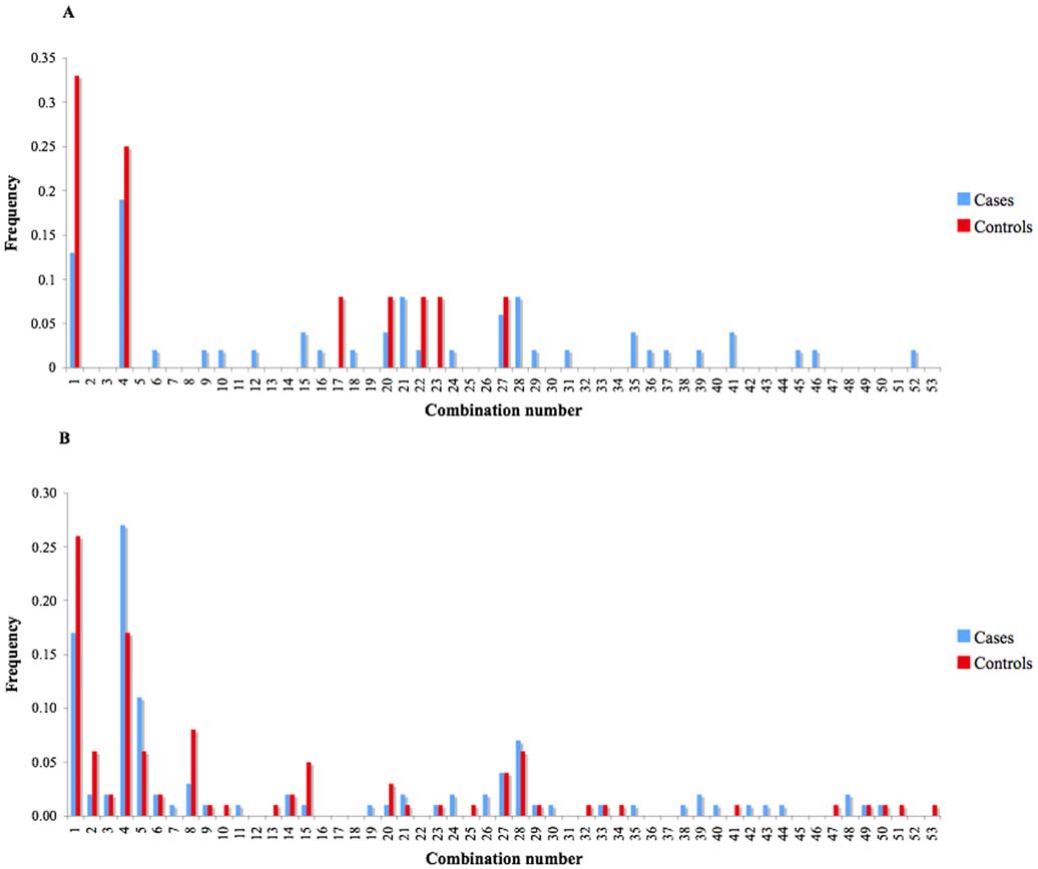
Combinations of SNPs in *TIRAP* found in individual patients or controls. Graph shows the frequency of 53 SNP combinations found in the A) Mixed ancestry and B) Xhosa groups. The blue bars are the cases and the orange bars are the controls. No combination of SNP was statistically significant between cases and controls when analysed by Fishers exact test.

To investigate whether individuals with combinations of *TIRAP* SNPs might influence disease occurrence, we undertook analysis of the frequency of combinations of two or more variants in cases and controls. There were more combinations in the Xhosa group than in the mixed ancestry group, which might be expected due to the small size of the mixed ancestry cohort. However, despite the small size of this group there were several combinations that were present that were not detected in the Xhosa. There were no statistically significant differences between cases and controls for any of the 53 combinations of SNPs that we observed in either ethnic group (table 3 and fig. 2)

### Effect of *TIRAP* variants on Disease Phenotype

We went on to investigated the role of TIRAP variants in relation to disease manifestations by comparing the frequency of each variant in patients with pulmonary compared to disseminated TB. No SNP showed any association with the combined phenotype of extrapulmonary TB compared to pulmonary TB in either ethnic group.

We further analysed 558C→T to see if this SNP was associated with TBM. No association was seen in the Xhosa cohort as there were only eight cases that had the heterozygous genotype and none of these had TBM. However, comparing TBM to all other forms of TB in the mixed ancestry cohort, a significant association of the heterozygous SNP was found (P<0.05, table 4). When we compared TBM to the controls this SNP was still significantly associated with disease (p = 0.02). There were in total 29 patients of mixed ancestry who had extrapulmonary TB. Of these, eight had the CT genotype and 6 of these had TBM. The other two had Pleural effusions and E. Nodosum.

**Table 4:** Genotype at position 558 of *TIRAP* and disease phenotype in the mixed ancestry cohort.

	CC	CT	TT	Total	
**Pulmonary**	21	3	1	25	
**Extrapulmonary**	20	8	0	28	
**Total**	41	11	1	**53**	NS
**TBM**	4	6	0	10	
**No TBM**	37	5	1	43	
**Total**	41	11	1	**53**	p<0.05
**TBM**	4	6	0	10	
**Controls**	13	2	1	16	
**Total**	17	8	1	**26**	p = 0.02

Numbers of patients with mixed ancestry who had extrapulmonary TB compared to those that had pulmonary TB (top panel) and patients that had TBM compared with those that had other forms of TB (middle panel) for each genotype at position 558 of *TIRAP*. The lower panel shows the number of TBM patients compared with the healthy controls.

### Cytokine levels and genotype

**Figure 3:**
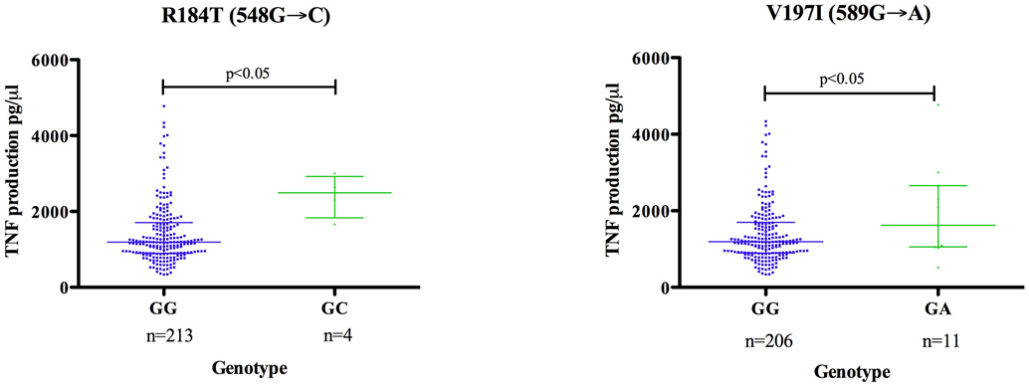
TNF induction in response to LPS in individuals with *TIRAP* variants. Graph shows the average TNF production in response to stimulation of whole blood with LPS from three separate experiments in Xhosa individuals with variant genotypes at positions 548 and 589 of the *TIRAP* gene. The error bars show the median with 95% confidence interval.

In order to establish whether any of the SNPs were associated with altered TLR signalling, as has been suggested in previous studies [20], we evaluated LPS induced TNF production as a marker of inflammatory gene induction through the TLR4 pathway from all controls and cases after they had cleared the infection. Although none of the identified SNPs showed significant difference in frequency between cases and controls, significant differences were seen in TNF production for two variants. In the Xhosa group we found that individuals carrying a single copy of the novel 548G→C (R184T) or the previously identified 589G→A (V197I) SNP, showed significantly higher TNF production than those with the wildtype (p<0.05) (fig. 3).

## Discussion

*TIRAP* is a critical gene in the TLR2 and TLR4 signalling cascades and recent studies have investigated its role in a number of infectious diseases including TB. There have been conflicting results from these papers, which may be due to ethnic differences in the populations studied. In order to understand the architecture of genetic variation across the gene, and its relationship to childhood TB, we screened the entire open reading frame for variation, rather than using only known variants. This approach allowed us to detect novel polymorphisms, as well as identify any disease-causing or rare mutations in either the control or disease populations, but did not detect intronic or promoter variants

We compared two ethnic groups from Cape Town, South Africa and showed that they have very different SNP patterns. Some SNPs were seen in both groups while others differed significantly in frequency. We identified several novel SNPs and observed a larger number (13 SNPs) than was found in studies on a Caucasian population (7 SNPs) [20] or a Vietnamese population (4 SNPs) [23]. Our data demonstrates the importance of identifying the pattern of variation in any ethnic population rather than relying only on previously reported frequencies of SNPs.

The 539C→T (S180L) has been shown to be protective against TB in a West African cohort [20]. This was confirmed in a small Columbian [21] study but was not reproduced in a joint Ghanaian, Russian, and Indonesian study [22] nor in a Vietnamese group [23]. This SNP was found at a frequency higher than 0.05 in the UK Caucasian population [20] but in all other studied populations, including ours, the frequency of the SNP was very low (we found the heterozygous genotype in only one case in the Xhosa group). The small sample size in our study is a major limitation when screening for low frequency SNPs. The 539T allele was only present at a frequency of 0.002 in this population and this may explain our inability to confirm the association of this SNP with protection from TB reported in other populations [21], [20] . However, what we can conclude is that due to its low frequency, any advantageous effect that this SNP confers will be of very limited significance in the Xhosa population.

There were significantly more heterozygous individuals in the mixed ancestry group compared to the Xhosa group but with the small numbers in our study we could not show significant association between genotype and disease. The high frequency of the SNP in European groups compared to African populations may be the consequence of selective pressure on *TIRAP*. However, in view of the complex selective pressure of several infectious diseases including TB, malaria, and bacterial sepsis, each of which may select different variants in TLR signalling, speculation on the reasons for ethnic variation in the genes controlling TLR signalling should await further data on the association of these variants with common childhood diseases in African and Caucasian populations.

Previous studies on a Vietnamese adult cohort have shown an association of the 558C→T heterozygous SNP with TBM [23]. This SNP was at very low frequency in the Xhosa group; however, in the mixed ancestry population the heterozygous variant was significantly more common in the subgroup with TBM. Six out of ten patients with TBM had the CT genotype whereas only one out of nine children with Pleural effusion had the CT genotype. The only other child to carry the CT genotype had E. Nodosum. These numbers are too small to draw any firm conclusions but they do suggest that the CT genotype may be associated with severer forms of TB particularly TBM and are consistent with the finding in the Vietnamese study.

The mixed ancestry group are a very heterogeneous population with many having East Asian ancestry [26]. This may explain why the SNP, which has been shown to affect TBM in Vietnamese, was more prevalent in this group compared with the Xhosa. It is also interesting to note that both the minor allele frequencies in SNP 539C→T and 558C→T were significantly different between the two ethnic groups studied. This again highlights the need to evaluate the whole gene when screening unstudied populations as opposed to searching only for previously published SNPs.

The 558C→T SNP does not cause an amino acid base change nor was it associated with altered TNF production in response to LPS in our study. This confirms the findings from the Vietnamese study that did not find an association with TNF production in response to LPS, which signals through the TLR4. However, they did find altered IL-6 production in response to lipopeptides, which signals through TLR2. This suggests that any effect of this SNP on *TIRAP* is specific to TLR2 signalling. The 558 SNP is however synonymous and may not itself be the disease causing allele, but may be in linkage disequilibrium with some other, unknown variant which we were unable to identify in our study. Alternatively the SNP may be having a function that we are unable to identify with our assays. Synonymous mutations have been shown to affect folding of mRNA secondary structures and thereby protein expression [27], [28] . Synonymous SNPs that occur close to the intron exon junctions can also disrupt the splicing process [29], [30], although this is unlikely to be the case with the 558 SNP as it is well within the exonic region. Although many non-coding SNPs have now been described that are associated with disease (for a list of diseases see [31]) the mechanism by which this SNP is associated with TBM at present remains unknown.

We found that the heterozygous SNPs 548G→C (R184T) and 589G→A (V197I), compared to the homozygous wildtype, were both associated with higher levels of TNF production in the Xhosa group. Neither of these SNPs was significantly more frequent in cases than controls suggesting that the increase in TNF in these individuals was not due to their having had TB. We measured TNF production in response to LPS stimulation, which acts via the TLR4 receptor [32], [33]. TLR2 rather than TLR4 is the predominant receptor triggered by TB [15], however, both receptors follow a similar signal transduction pathway requiring TIRAP. Individual polymorphisms in *TIRAP* may affect signalling of each of the TLRs differently and so the increase in TNF production following LPS stimulation may only be relevant to TLR4 and may not affect TLR2 signalling in the same way. These functional assays would have been better carried out using a TLR2 agonist or stimulant. At the time of enrolment to our study *M.Tb* was thought to activate both the TLR2 and TLR4 pathways [13], and as the functional assay using LPS was used to probe interferon gamma responses, and both TLR2 and TLR4 followed the same pathway downstream of TIRAP, it was reasoned that LPS stimulation would be sufficient to demonstrate the function of TIRAP in TB. Since then it has been shown that it is TLR2 that is directly involved with TB immunity [15], and it may have been preferable to use both TLR2 and TLR4 agonists to explore the role of *TIRAP*.

In order to avoid the confounding effect of ethnic difference, we kept the Xhosa and the mixed ancestry cohorts separate throughout this study. This resulted in small numbers in the individual cohorts, particularly in the mixed ancestry group. Large numbers are required to demonstrate moderate effects of low frequency SNPs. While the low numbers of the 539C→T SNP in the Xhosa population makes it difficult to draw definite conclusions on the role of this variant, our data may in future be used in meta-analysis in combination with other childhood cohorts to confirm the association

The mixed ancestry population are an admixed group and very large numbers of samples would be required to eliminate “ethnic specific” confounders and to be able to draw significant conclusions. Despite the small numbers the significant association of the 558 SNP with TBM is in keeping with the Vietnamese study and is thus likely to represent a genuine effect.

Our work is the first study to investigate the role of polymorphisms in the TIRAP gene in a paediatric cohort. Our results suggest that polymorphic variation in this gene is not involved in susceptibility to TB in Xhosa children or those of mixed ancestry in South Africa, but may affect the likelihood of developing TBM. This is particularly interesting as children are more likely to develop TBM than adults Further work on the relevance of this gene in a larger cohort would therefore be beneficial. Furthermore the markedly different SNP pattern in the two ethnic groups highlights the importance of identifying SNPs present in different ethnic populations first, rather than utilising only published variants from case control studies, before drawing conclusions about disease association

## Methods

### Ethics statement

This study was conducted according to the principles expressed in the Declaration of Helsinki. The study was approved by the Research Ethics Committee, University of Cape Town and St. Mary's London (LREC: 01/GB/218E; Reference number: 013/2000). All patients provided written informed consent for the collection of samples and subsequent analysis.

### Study subjects

Children of Xhosa origin and mixed ancestry were recruited at Red Cross Children's hospital in Cape Town, South Africa. The Xhosa are an indigenous black population of South Africa and speak the Xhosa language. Those of mixed ancestry are from the group commonly referred to in South Africa as Coloureds. These people are from a mix of several racial ancestries from Europe, Indonesia, Madagascar and from across Southern Africa. The Cape Coloureds make up the predominant population group in the Western Cape and are of Malay origin mixed with either European or the indigenous Khoi and San people. They are referred to throughout the paper as mixed ancestry.

Cases were drawn from two studies, one of acute newly diagnosed TB, and one of patients previously treated for TB (Past history cases). Acute cases were recruited at the time of admission and the commencement of anti-TB chemotherapy, while past history cases were enrolled at least one year after their initial diagnosis. They were identified via the hospital and microbiology departmental records of The Red Cross Children's Hospital, Cape Town. In the Xhosa group 83 children were enrolled as acute cases and 59 were enrolled as past history cases. In the mixed ancestry group 42 were acute cases and 12 were past history cases. Cases were categorised according to the certainty of their TB diagnosis. Those categorised as definite TB had a diagnosis confirmed by culture of *Mycobacterium tuberculosis (M. tb)* from sputum, gastric aspirate, CSF or other body fluids or biopsy material (Xhosa n = 44; Mixed ancestry n = 14). Highly Probable TB had Acid Fast Bacilli (AFB) identified in sputum, gastric aspirate, CSF, other body fluids or on histological examination of biopsy material but with no culture confirmation having been obtained. (Xhosa n = 8). Probable TB was diagnosed according to World Health Organisation (WHO) criteria for suspecting TB in children (Xhosa n = 90, Mixed ancestry n = 40). The clinical records, and microbiological results of all patients were reviewed by two paediatricians with experience of childhood TB, and only those in whom the findings and response to treatment was consistent with TB were retained in the study.. Children over 14 years of age, HIV seropositive or with other underlying immunodeficiency or those that had been on prolonged treatment with steroids or other immuno-suppressive drugs were excluded from the study.

Neighbourhood, age matched controls comprised of 122 Xhosa children and 16 of mixed ancestry. These controls were living within three streets of, and were unrelated to a case. Mantoux skin testing for evidence of infection with *M. tb* was carried out by one nurse, using 2 tuberculin units of purified protein derivative RT-23 [Statens Serum Institut, Copenhagen, Denmark]. Controls were categorised as Mantoux positive if they had an induration diameter of ≥10 mm. Any child with an induration diameter of 15 mm or greater was referred to the local TB clinic for chest x-ray and assessment in accordance with national guidelines and excluded from analysis if diagnosed with TB. Clinical data was obtained for all patients and blood collected for functional assays and DNA extraction.

All subjects in the study lived in townships in Cape Town both in formal and informal housing. These townships are very crowded and over two thirds of the controls and 70% of cases had been in contact with someone with TB, often a household member.

As we were dealing with a paediatric group, it is always possible that a control could become a case in future. All controls and past history cases were therefore seen again one month and 6 months after their initial visit to confirm that their status had not changed.

### Functional studies to measure TNF upregulation in whole blood

Blood from all study subjects was collected at recruitment and on up to three consecutive visits (Admission, 6 months and 1 year for acute cases, and enrolment, one month and six months for all others). For the assessment of TNF responses, only data obtained at least 6 months after commencing anti TB treatment was utilised

Blood was collected into preservative-free heparin tubes was diluted 1∶10 in RPMI 1640 (supplemented with penicillin and streptomycin and L-glutamine). A round bottomed 96 well plate containing 180 µl of the diluted blood was set up to include 4 different experimental conditions. These included wells with media only or with 0.5 ng/ml. The LPS was added only after the cells had been allowed to incubate for two hours at 37°C in a 5% CO_2_ humidified incubator. Each condition was carried out in quadruplicate and plates incubated overnight. Supernatants were harvested after 24 hours and the concentration of TNF determined by standard ELISA methods. All cytokine assays were carried out on the day that the blood was collected.

### Amplification of the TIRAP open reading frame and 3′ untranslated region by PCR

Primers spanning the two coding exons of *TIRAP* were synthesized by Sigma genosys (Haverhill, UK). Exon 1 primers were as previously published [23]. For mutation detection gel analysis (MDGA) optimal product size was 300 bp so PCR amplification of exon 2 was split into 2 regions. Exon 2i was analysed by MDGA using the forward primer CTCTGAGAATAAGATGTTTCC and the reverse primer ACGCAGACGTCATAGTCTTT. Exon 2ii had too many SNPs to be detected by this method and was analysed by direct sequencing. The 2ii forward primer was AGTGACAGTGGCAGTAGTC and 2ii reverse primer was CCTGTTGGTCAGTGAGGAAA. Primers were designed with both 3′ Fam labelling for use in MDGA and without any label for sequencing. Standard PCR reactions were carried out using 1.5 µg/µl MgCl_2_ and an annealing temperature of 59°C for exon 1 and 2i and 64°C for exon 2ii.

### Mutation Detection Gel Analysis (MDGA)

MDGA is a high throughput method which combines single stranded conformational polymorphism (SSCP) and heterduplex mutation analysis (HMA) to identify sequence changes by shift in the electrophoretic migration of the DNA [34] The entire cohorts of patients and controls were screened for sequence variation, with any band shifts being confirmed by sequencing.

One microlitre of the PCR product was added to 3 µl of Rox 500XL (Applied Biosystems, Warrington, UK) and Elliomut loading buffer (Ellios biotek Laboratory, Paris, France), denatured at 96°C for 2 minutes and cooled immediately on ice. Samples were then loaded onto 36 cm Elliomut gels (Ellios biotek Laboratory, France) using 96 well CAM combs and run for 3.5 hr on the recommended manufacturers programme for MDGA. The Elliomut gel is a specially formulated non-denaturing polyacrylamide gel that allows the separation of single stranded DNA by conformational changes and also analysis of the heteroduplex. Samples were analysed using the ABI 377 using Genescan and Genotyper programmes. All bandshifts and pattern changes (i.e. migration profiles) were recorded. Random samples showing different bandshifts were PCR amplified with unlabelled primers, cleaned, cycle sequenced and then run on a capillary sequencer at the University of Oxford, Department of Zoology sequencing facility.

### Statistical Analysis

Differences between cases and controls for each detected SNP and between combinations of SNPs were investigated by Fishers exact test using Graphpad prism.

Data plotted for TNF production for each individual was the average result of functional assays performed on the six and 12 month visit for acute cases, and the average of three visits for the past history cases and controls. Differences in TNF production between different genotypes was analysed by the Mann Whitney t test where only two genotypes were present, or the Kruskall Wallis ANOVA when all three genotypes were identified.
